# Metabolic maturation in the infant urine during the first 3 months of life

**DOI:** 10.1038/s41598-024-56227-7

**Published:** 2024-03-08

**Authors:** Julie Astono, Katrine O. Poulsen, Rikke A. Larsen, Emma V. Jessen, Chatrine B. Sand, Morten A. Rasmussen, Ulrik K. Sundekilde

**Affiliations:** 1 Department of Food Science, Aarhus University, Agro Food Park 48, Aarhus N, Denmark; 2 Sino-Danish Center, Niels Jensens Vej 2, Building 1190, Aarhus, Denmark; 3 Department of Food Science, University of Copenhagen, Rolighedsvej 26, Frederiksberg, Denmark; 4 COPSAC, Herlev-Gentofte Hospital, Ledreborg Alle 28, Gentofte, Denmark

**Keywords:** Metabolomics, Paediatric research

## Abstract

The infant urine metabolome provides a body metabolic snapshot, and the sample collection can be done without stressing the fragile infant. 424 infant urine samples from 157 infants were sampled longitudinally at 1-, 2-, and 3 months of age. 49 metabolites were detected using proton nuclear magnetic resonance spectroscopy. Data were analyzed with multi- and univariate statistical methods to detect differences related to infant age-stage, gestational age, mother’s pre-pregnancy BMI, C-section, infant birth weight, and infant sex. Significant differences were identified between age-stage (p_bonferoni_ < 0.05) in 30% (15/49) of the detected metabolites. Urine creatinine increased significantly from 1 to 3 months. In addition, myo-inositol, taurine, methionine, and glucose seem to have conserved levels within the individual over time. We calculated a urine metabolic maturation age and found that the metabolic age at 3 months is negatively correlated to weight at 1 year. These results demonstrate that the metabolic maturation can be observed in urine metabolome with implications on infant growth and specifically suggesting that the systematic age effect on creatinine promotes caution in using this as normalization of other urine metabolites.

## Introduction

Metabolomics is a relatively new analysis method in which all metabolites in a sample under certain conditions, also known as the metabolome, are identified, and possibly quantified. Metabolites give a snapshot of the metabolic state of the body because they are products of the ongoing processes in the body that work to maintain the metabolic state^1^. Consequently, metabolomics holds a great potential for being used for diagnostics of different diseases if a change in the metabolic processes occurs^2,3^. To take advantage of metabolomics clinically, it is necessary to know the reference values of healthy individuals, which are one of the specific aims of this study. The two other specific aims of the study were to (1) determine if the infant urine metabolite levels differ when related to infant age-stage, gestational age, mother’s pre-pregnancy BMI, C-section, infant birth weight, and infant sex. (2) Determine a metabolic maturation age based on the urine metabolite levels. The urine metabolome is obvious to investigate as the infant is not stressed at the collection of the sample compared to collecting a blood sample^4^. Normally, screening of infants for inborn errors of metabolism is done from a blood spot taken from the heel. However, infant urine metabolomics analyzed with proton nuclear magnetic resonance spectroscopy (^1^H NMR) holds great potential for diagnosing inborn errors of metabolism in a non-invasive, time efficient, and economically preferable way^2^. Furthermore, there is a gap in the understanding of the infant urine metabolome compared to the adult urine metabolome. More than 2000 metabolites have been detected and quantified in adult urine^5^, for which only a minor fraction (n = 378 public available via the Human Metabolome Database (HMDB)^6^) have been detected and quantified in infant urine^7^. Previous studies on the infant urine metabolome are limited, cross sectional and small in sample size^7–9^. Hence, they do not obtain information on the development of the metabolome and the possible reflections happening in the body during this phase of rapid growth. Large discrepancies between the studies are also observed in mean concentrations. Some studies obtain differences in the infant urine metabolome between sex, while others see no difference^7,8^. However, limited literature about infant urine metabolomics is available emphasizing the lack of research in this field. Determination of maturity of the child based on other measures, than direct clinical data is known from the gut microbiome as the “relative microbiota maturity index”. This index can be used to estimate the malnourished states in children, based on the degree of gut microbiome maturity^10^. Urine metabolites have also been used to determine the metabolic age of stunted children^11^. However, we hypothesize that we can use the obtained data of urine metabolites from 1, 2, and 3 months to determine a metabolic maturation age of healthy children as well. We aim to correlate the metabolic maturation age to prospective outcomes at 1 year. This analysis will aid in the understanding of which metabolites in the early months are important drivers for growth in the first year of life.

In this study, we investigate the infant urine metabolome from samples collected at 1, 2, and 3-months of age on a total of 424 urine samples (n = 157 infants). This is, to date, the largest longitudinal study of infant urine metabolites. We use ^1^H NMR to analyze the samples, as this method provides precise and robust results. Furthermore, the sample preparation is simple and high-throughput, compared to mass spectrometry, which is the other preferred method used for analyzing metabolites^12^.

## Materials and methods

### Participants and sample collection

Infants providing samples for this study were recruited as part of the MaInHealth cohort established in Aarhus, Denmark^13^. The MaInHealth project investigates the natural human milk variation and the possible effects on infant metabolism and gut microbiota. The pregnant women were recruited from Aarhus University Hospital, Aarhus, Denmark from 2019 to 2021. Informed consent was obtained from women involved in the study and from both parents on behalf of the infant in accordance with the Declaration of Helsinki II. Ethical approval for this study was granted by The Central Jutland Regional Committee on Health Research Ethics (journal number 1-10-72-296-18v6). The study is registered at ClinicalTrials.gov, identification number: NCT05111990. Women included in the study were healthy, non-smokers, expecting to give birth vaginally, and expecting to breastfeed for the first 4 to 6 months. Infants included were healthy, with average birth age of 2500–5000 g and were born full-term (minimum 37 weeks of gestation). For a detailed description of the project, recruitment, and sample collection, see the study protocol: Influence of maternal body mass index on human milk composition and associations to infant metabolism and gut colonization: MAINHEALTH—a study protocol for an observational birth cohort^13^.

Briefly, infant urine was collected by placing a cotton pad in the infant’s diaper at 1, 2, or 3 months post-partum. After urination, the cotton pad was transferred to a Corning Gosselin Screw Cap 40 mL Container tube (Corning, New York, USA) and stored in the participants’ own freezer at − 20 °C until collection. Samples were gathered within 14 days. Storage at − 20 °C for 14 days should not affect the metabolic fingerprint of the urine sample^14^. The samples were transported on dry ice to Department of Food Science, Aarhus University, where they were stored in − 80 to − 70 °C until further analysis.

### ^1^H nuclear magnetic resonance spectroscopy metabolomics analysis of infant urine

The cotton pads containing the infant urine were removed from the container tube and thawed at room temperature. 50 mL tubes were prepared with three closed 2.0 mL Eppendorf tubes in the bottom. These Eppendorf tubes were used to withhold the cotton pad, while the urine was centrifuged to the bottom of the 50 mL tubes at 2000×*g* for 5 min at 4 °C. The cotton pads and Eppendorf tubes were discarded. 630 μL of the extracted urine and 70 μL phosphate buffer (1.5 M KH_2_PO_4_, 2 mM NaN_3_, 6 mM 3-(trimethylsilyl)propionic-2,2,3,3-d_4_ acid (TSP) in D_2_O, pH = 7.4) were transferred to a 1.5 μL Eppendorf tube and vortexed for 30 s and centrifuged shortly for 5 s. 600 µL of the prepared samples and buffer were transferred to 5 mm NMR tubes and cleaned before they were subjected to NMR analysis.

The samples were analyzed on a Bruker Avance Neo 600 spectrometer equipped with a BBI Probe (Bruker BioSpin, Rheinstetten, Germany). ^1^H NMR spectra were acquired using Bruker’s In Vitro Diagnostics research (IVDr) platform for automated metabolite quantification in body fluids with the B.IQuant-UR1.1 module, which is used for urine metabolite quantification^2^. Briefly, a 1D ^1^H spectrum using Bruker pulse sequence 'noesygppr1d' was acquired with 32 scans, using 4 s delay, 20 ppm spectral width, and 64 K complex data points. FID acquisition time was 2.7 s. Prior to Fourier transform, a 0.3 Hz linebroadening factor was applied and the FID zero filled to 128 K data points. The obtained spectrum was automatically phase and baseline corrected and referenced to TSP signal. Finally, an artificial PULCON reference signal with an amplitude corresponding to 1 mM ^1^H concentration was added at 12 ppm to enable a quantitative spectrum analysis^2^.

In the IVDr standard operating procedure a 2D JHH resolved spectrum using ‘jresgpprqf' was acquired with 2 scans, 2 s delay, 16.7 ppm sweep width, 8 K complex data points. The FIDs were multiplied with a sine function and zero filled to 16 K in the direct and 256 in the indirect dimension prior to Fourier transform. The obtained spectrum was processed in magnitude mode and tilted (45°) to construct a homodecoupled ^1^H spectrum by projection in the direct dimension^2^.

### Identification of metabolites and statistical analysis

To ensure correct metabolite identification, quantification, and to identify additional metabolites in the infant urine, besides the ones in determined by IVDr (Bruker Biospin), the acquired 1D spectra were analyzed using Chenomx NMR suite 9.0 (Chenomx Inc., Edmonton, AB, Canada) with the Chenomx standard metabolite library and an in-house metabolite library. The spectra were referenced to TSP signal at 0 ppm. Metabolite concentrations were normalized to the total mean metabolite concentrations in each urine sample prior to analysis^15^.

Multivariate analysis was performed using SIMCA 17 (MKS Data Analytics Solutions, Umea, Sweden). Principal component analysis (PCA) was used to inspect the dataset for outliers and variation according to age-stage. The metabolite concentrations were Unit Variance-scaled prior to the analysis by division with the standard deviation. Potential outliers were identified by inspecting the Hotellings T^2^ plot with a cutoff point at 95%. Univariate analysis was performed using R statistical software (4.3.0). Effects of infant age-stage, gestational age, mother’s pre-pregnancy BMI, C-section, infant birth weight, and infant sex on urine metabolites levels were analyzed using Linear mixed-effects models^16^. Correction for multiple testing was performed by Bonferroni^17^.

### Establishing metabolic maturation

A partial least squares (PLS) regression model was built (SIMCA 17) with sample age in days as the response variable and metabolite levels as the explanatory variables from all samples, including cross validation to avoid overfitting. Confidence intervals of the metabolite coefficients in the PLS-model were based on the internal PLS cross validation models. The metabolic maturation is defined as the difference between the actual age and the prediction solely based on the metabolite profiles in the urine.

Correlation between the predicted metabolic maturation age and the prospective outcomes weight at 1 year and height at 1 year was analyzed using a linear model in R statistical software (4.3.0). The model was corrected for infant sex, birth weight, and gestational age.

## Results and discussion

### Subject characteristics

157 mother-infant dyads are included in the MaInHealth cohort, and the subject characteristics are given in Table 1. This study was performed on 424 samples of infant urine divided between the 3 months (Table 1). However, not all infants gave samples to all time points and this study included 118 participants with full completion. The average pre-pregnancy BMI of the mothers was 26.59 kg/m^2^ (Table 1). The high average pre-pregnancy BMI is due to the purpose of the MaInHealth cohort, which is to investigate differences in human milk between normal weight and overweight/obese mothers and the possible outcomes on infant metabolism and gut microbiota. Planned birth by C-section was an exclusion criterion, whereas emergency C-section after rupture of membranes was allowed. 13 of the mothers gave birth by C-section, which corresponds to 8.28% of the cohort (Table 1). There was an almost equal distribution of sexes between the infants with 51.52% of the infants being female (Table 1).

**Table 1:** Sample characteristics of the cohort.

Samples at 1/2/3 months (n)	137/147/140
Samples with full completion (n)	118
GA at birth (weeks)	40.44 ± 1.08
Mother's pre-pregnancy BMI (kg/m^2^)	26.59 ± 5.19
C-section (n)	13
Infant birth weight (g)	3749.36 ± 444.70
Infant sex (female/male)	81/76
Weight at 1 year (kg)	9.97 ± 1.79
Height at 1 year (cm)	75.36 ± 9.64

Continuous data presented as means ± standard deviation. Categorical data are presented as numbers included in each category.

BMI: body mass index, C-section: Cesarean section, GA: gestational age, n: number.

### Infant urine creatinine levels change during the first 3 months of life

Urine creatinine levels are often used to normalize metabolomics data, thereby accounting for dilution effects. This practice is based on studies that have determined that urine creatinine levels are nearly constant in individuals and independent of fluid intake^18,19^. However, our results indicate that this is not the case for infants during the first 3 months of life. We observed a significant increase in the infant urine creatinine level over the first 3 months from 1321.54 ± 443.36 µmol at 1 month to 1696.06 ± 430.03 µmol at 3 months (Table 2). The raw concentrations of creatinine were 1349.98 ± 1002.90 µmol, 1401.23 ± 1278.62 µmol, 1923.91 ± 1774.47 µmol at 1, 2 and 3 months, respectively. We did not observe a difference in urine creatinine levels between infant sexes (Fig. S1 and Table S1 in supporting information). Consequently, the application of creatinine normalization could potentially yield erroneous outcomes. Accordingly, metabolite concentrations in our study were normalized to total metabolite concentrations in each urine sample prior to analysis, as commonly applied normalization factor in urine metabolomics^15^.

**Table 2:** Mean concentrations of infant urine metabolites ± standard deviation from 1-, 2-, and 3-months.

	Concentration (µmol) ± SD		
	1 month	2 months	3 months
Amino acids and derivatives			
Alanine	399 ± 92	413 ± 99	405 ± 99
Betaine	1598 ± 383	1622 ± 361	1550 ± 389
Carnitine	38 ± 30	40 ± 32	43 ± 34
Cysteine	269 ± 137	244 ± 137	277 ± 140
** Glutamine**	330 ± 110^a^	405 ± 124^b^	420 ± 124^b^
** Glycine**	1383 ± 440^a^	1119 ± 426^b^	957 ± 365^c^
Glutathione	39 ± 13	36 ± 16	41 ± 16
Guanidoacetate	187 ± 92	185 ± 53	184 ± 61
Lysine	96 ± 46	97 ± 45	85 ± 43
Methionine	45 ± 25	42 ± 25	39 ± 24
** N,N-Dimethylglycine**	221 ± 95^a^	193 ± 64^b^	193 ± 89^b^
** N-Acetyltyrosine**	23 ± 33^a^	44 ± 69^ab^	50 ± 59^b^
** Sarcosine**	13 ± 5^a^	10 ± 4^b^	7 ± 3^c^
Serine	513 ± 161	560 ± 179	543 ± 185
Taurine	741 ± 267	785 ± 275	789 ± 301
Threonine	272 ± 96	265 ± 91	247 ± 68
Valine	57 ± 22	58 ± 28	55 ± 27
Energy related			
** 2-Oxoglutarate**	240 ± 194^a^	192 ± 139^ab^	173 ± 105^b^
Acetate	555 ± 512	630 ± 736	524 ± 546
** Citrate**	1923 ± 817^a^	2069 ± 772^a^	2471 ± 1062^b^
** Acetoacetate**	12 ± 9^a^	14 ± 8^a^	17 ± 9^b^
Acetone	39 ± 21	36 ± 17	35 ± 24
Creatine	108 ± 110	111 ± 144	164 ± 197
** Creatinine**	1310 ± 365^a^	1551 ± 345^b^	1696 ± 430^b^
Formate	1222 ± 807	1264 ± 641	1261 ± 716
Fumarate	17 ± 9	15 ± 7	15 ± 10
Homocysteine	435 ± 314	465 ± 135	503 ± 249
Lactate	216 ± 318	231 ± 421	196 ± 342
Pyruvate	41 ± 36	38 ± 26	35 ± 26
Succinate	117 ± 56	113 ± 45	111 ± 47
Fatty acids and derivatives			
** Choline**	56 ± 40^a^	45 ± 22^b^	40 ± 16^b^
** S-Adenosylhomocysteine**	6 ± 2	6 ± 2	6 ± 2
Not classified			
** 1-Methylnicotinamide**	104 ± 37^a^	88 ± 44^ab^	81 ± 59^b^
Allantoin	72 ± 20	73 ± 22	66 ± 24
Pantothenate	57 ± 19	60 ± 19	56 ± 20
O-Phosphoethanolamine	142 ± 51	136 ± 44	134 ± 39
Phenylacetate	55 ± 18	62 ± 51	59 ± 34
Propylene glycol	1966 ± 1517	2042 ± 1468	2081 ± 1836
Food derived			
Caffeine	55 ± 35	59 ± 36	66 ± 43
Dimethylamine	317 ± 71	330 ± 102	311 ± 122
** Tartrate**	58 ± 21^a^	37 ± 21^b^	24 ± 14^c^
** Trimethylamine**	5 ± 2^a^	7 ± 2^b^	8 ± 2^c^
** Hippurate**	138 ± 90^a^	159 ± 73^ab^	181 ± 95^b^
Sugars			
Galactose	884 ± 356	840 ± 301	848 ± 312
Glucose	391 ± 232	376 ± 254	371 ± 195
Lactose	722 ± 270	711 ± 243	678 ± 237
Mannose	27 ± 47	24 ± 54	12 ± 28
** Myo-inositol**	951 ± 398^a^	563 ± 272^b^	357 ± 180^c^
Microbial origin			
Indole-3-lactate	14 ± 16	16 ± 25	20 ± 24

Concentrations are represented as µmol after normalization to mean metabolite content. Metabolites in bold represent statistically significant difference calculated with linear-mixed effects models after Bonferroni correction (p < 0.05).

Different studies have determined that the level of urine creatinine increases until around 20–29 years of age depending on the sex^8,20^. Most of these studies have been performed on children or adults, and few studies focus on infants despite large metabolic developments in the first months of life. A similar study by Scalabre et al. investigated the urine metabolome from zero to 4 months of age on 90 infants in total, however the age-stages consisted of different participants meaning that all infants only provided one sample^8^. They did not find a change according to age but did find a change according to weight, which was not the case in this study. Creatinine production and excretion in adults depend on the glomerular filtration rate, muscle mass and intake of especially red meat^19^. Red meat intake is, however, absent in breast-fed infants. The difference in urine creatinine levels according to age are likely to be attributed to a development in the muscle metabolism^21,22^. These results highlight the importance of utilizing age-specific creatinine reference values, which could be established based on age-stages, such as monthly intervals.

It is well-known that the urine creatinine production and thereby excretion is different between adult males and females^20,23^. Adult males have around 36% more muscle mass than females^24^. In infants that difference is only around 8%, which could explain the lack in difference between sexes in urine creatinine excretion among infants^25^.

### Urine metabolites changes with infant age

**Figure 1:**
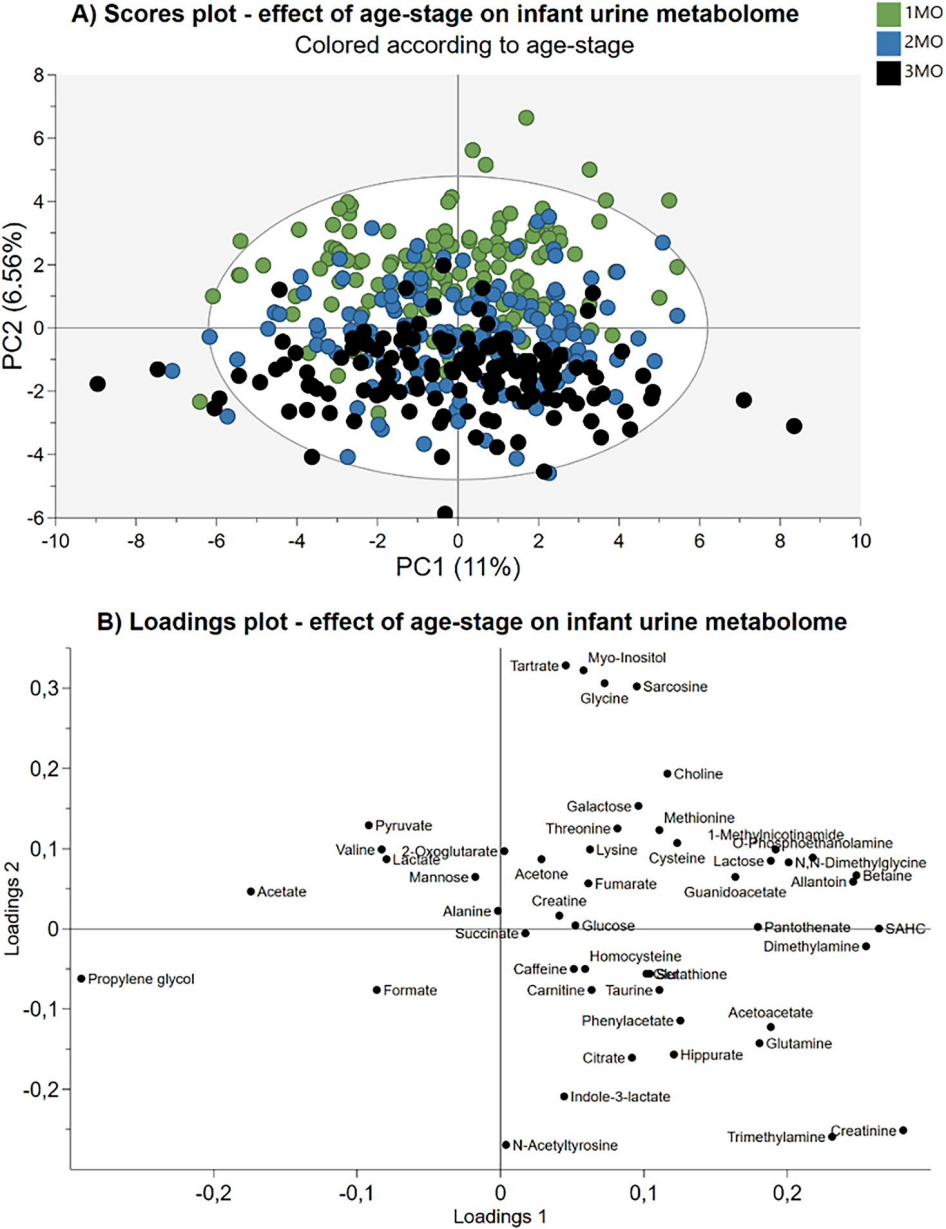
Principal component analysis of infant urine samples (n = 422) from 1-, 2-, and 3-months samples. (**A**) Scores plot of samples colored according to age-stage showing principal component 1 on the x-axis and principal component 2 on the y-axis. Legend denotes color codes, MO: Month. (**B**) The corresponding loadings plot of the measured variables. SAHC: S-Adenosylhomocysteine, Ser: Serine.

A total of 49 metabolites were identified and quantified in the 424 samples using IVDr and Chenomx in combination (Table 2). The metabolites included 17 amino acids and derivatives, 13 energy related metabolites, 5 carbohydrates, 2 fatty acids and derivatives, 5 food related metabolites, and 1 microbiota related metabolite. 6 metabolites were not classified (Table 2). A principal component analysis (PCA) model was based on the metabolite levels in all samples to identify outliers and detect grouping and separation trends (Fig. 1). The PCA model showed 2 distinct outliers potentially influencing the overall variation explained by the model, these 2 samples were therefore excluded from this and all future analyses. The cotton pad from where the sample was extracted was contaminated with feces during the collection from the diapers. The PCA model consisted of 4 principal components (PCs) and explained 27.9% of the variation in the data.

Figure 1A shows PC2 plotted against PC1, and it explains 17.56% of the variation. The samples from 1 month are mainly located in the upper part of the plot, while the 3-months samples are located in the bottom part. The 2-months samples are located around the center of the plot overlapping between the 1- and 3-months samples (Fig. 1A). Figure 1B shows the corresponding loadings plot. Myo-inositol, sarcosine, tartrate, and glycine are found in the upper part of the loadings plot and is positively correlated to 1-months samples. Opposite, creatinine, trimethylamine, and N-acetyltyrosine are found in the lower part of the plot, hence are negatively correlated to the 1-month samples. Furthermore, choline seems to be positively correlated to 1-month samples and citrate, hippurate, indole-3-lactate, acetoacetate and glutamine are found to be negatively correlated to the 1-month samples.

When plotting PC2 against PC3 and PC4, respectively, infant age explains some of the variation in these components (Figs. S2 and S3 in Supporting Information).

All infants included in the study received human milk as their primary feed. The metabolite content of the human milk changes during the lactation even after 1 month, where it has translated into mature milk^26^. This will add complexity to the discussion regarding the biological relevance of the excreted metabolites. The excretion of metabolites is not only dependent of the intake but also of the kidney and the glomerular filtration rate, which has not reached its full capacity at the age of 3 months. The rate increases until the age of 2 years^27^. In combination with that, the infant body and metabolism develop throughout the 3 months leading to changes in urine metabolite content.

### Linear mixed-effects model analysis of metabolite concentrations

**Figure 2:**
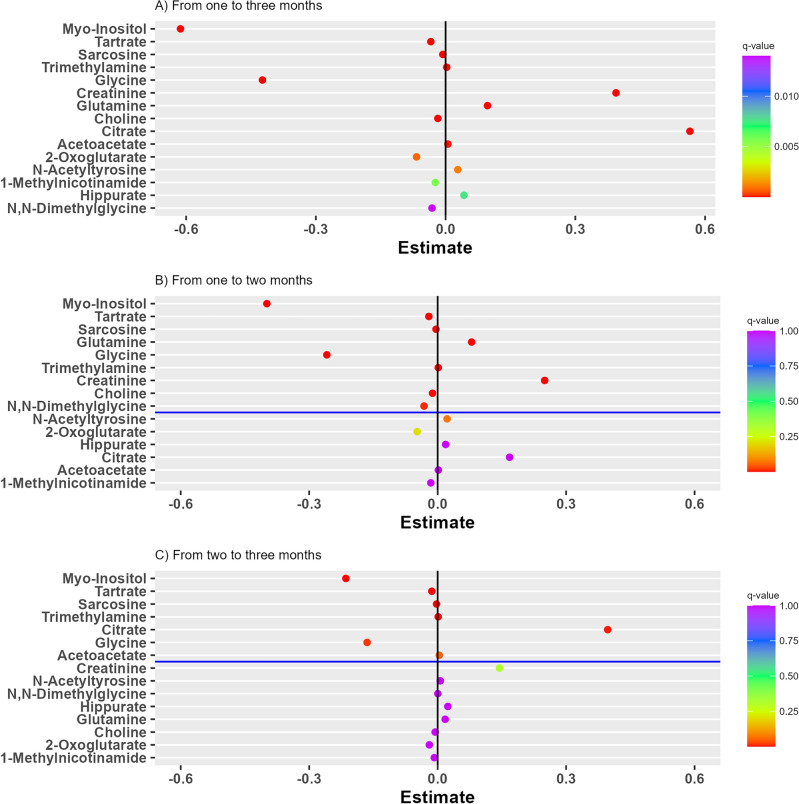
Forest plots of linear mixed-effects models of metabolite levels from (**A**) 1 to 3 months, (**B**) 1 to 2 months, and (**C**) 2 to 3 months. Figures display the −log10(q-value) versus the β-estimates with statistical significance from 1 to 3 month at a significance level of 0.05 corresponding to –log10(q-value) = 1.30 after Bonferroni-adjustment marked with a blue line.

A univariate statistical analysis was conducted to assist the multivariate analysis of the data and to determine the statistical relation between the measured metabolites and the explanatory variables. To account for the repeated measurements from the same individual, the data was analyzed using linear mixed-effects models for each metabolite. The fixed-effects included in the model were infant age-stage (1-, 2-, or 3-months), gestational age, mother’s pre-pregnancy BMI, C-section, infant birth weight, and infant sex. 15 of the 49 detected metabolites differed between age-stage displayed significant β-estimates in the linear mixed-effects models (Fig. 2 and Table 2). The results of the metabolites related to age-stage are shown in the forest plots in Fig. 2. Results for linear mixed-effects models of all metabolites including fixed and random effects can be found in Supporting Information (Tables S1 and S2).

The low β-estimates and a q-value < 0.0001 of myo-inositol, indicates that it has the largest decrease in infant urine from 1 to 3 months of age among all the measured metabolites (Fig. 2). Other metabolites with a significant negative β-estimate from 1 to 3 months include 1-methylnicotinamide, 2-oxoglutarate, choline, glycine, N,N-dimethylglycine, sarcosine, and tartrate (Fig. 2). Metabolites with a positive β-estimate from 1 to 3 months include acetoacetate, citrate, creatinine, glutamine, hippurate, N-acetyltyrosine, and trimethylamine (Fig. 2). All metabolites with significant β-estimate from 1 to 3 months also included those with significant β-estimate between 1 and 2 months, and 2 and 3 months. The increase and decrease in the excreted metabolites from the infants indicate a clear correlation to the development in the infant during the postnatal period.

Urine glutamine level from 1 to 3 months increased. Glutamine is a non-essential amino acid imporatant in multiple pathways, including proteins and nucleotide synthesis^28^. It acts as a precursor of glutamate and, through that, alanine, providing energy for the tricarboxylic acid (TCA) cycle particularly important for the intestinal tissue^28^. Furthermore, glutamine is involved in the regulation and interaction of the immune system^29^. A reason for the increase in urine glutamine over time could also be attributed to intake, as the content increases in human milk with time, potentially saturating the infant with glutamine^30,31^.

The rapid infant growth in the initial months requires much energy with 35% of the energy intake allocated for growth the first 3 months of life^32^. Notably, several of the metabolites that change over time in this study are implicated in energy metabolism, particularly the TCA cycle. Citrate, a TCA cycle constituent, synthesized from acetyl CoA and oxaloacetate^33^ shows an increase in urine content over time, despite a decrease in human milk^30,31^. This could suggest improved citrate utilization efficiency or an alternative synthesis process. Conversely, 2-oxogluterate, another TCA cycle component, also known as alpha-ketoglutarate decreases over time^33^. Potentially indicating an increased energy requirement or utilization in another process in infants. Acetoacetate, a ketone body, is converted to acetyl CoA, from where it enters the TCA cycle. Some parts of the tissue prefer to use ketone bodies as energy source over glucose, for example the heart muscle^33^. The observed increase in acetoacetate urine levels over time, could indicate a reduced utilization of acetoacetate as an energy source, potentially reflecting a metabolic shift in infants.

Glycine is involved in many different processes, including creatine production, together with arginine and methionine^34,35^. We observe a decrease in glycine excretion, but we see a tendency for creatine increase. Elevated urine creatinine, but also creatine, may indicate growth, correlating with an increase in muscle mass. Creatine production is not the only process glycine is involved in. Glycine is also involved in the one carbon metabolism (OCM), linking different pathways ensuring transfer of 1C moieties to metabolic processes as necessary. These processes include protein synthesis, nucleotide synthesis, and epigenetic regulation of DNA with methylation among others. The OCM is known to be important for the infant as many of the processes the OCM supports are fundamental for growth and development^36^. Glycine, choline, sarcosine, and dimethylglycine contribute to the OCM by primarily donating 1C units to tetrahydrofolate, which act as a carrier of activated 1C units. The observed decrease in urine levels for these metabolites suggest higher utility, thereby supporting rapid growth and development experienced in the infant.

Myo-inositol is a cyclohexanehexol and can also be synthesized from glucose-6-phosphate^37^. Myo-inositol is attached covalently to phospholipids to yield phosphatidylinositols. Phosphatidylinositols, essential constituents of cell membranes, and secondary messengers, also play a role in the development of the infant brain^38^. Its metabolism primarily takes place in the kidney and studies in rodents show that the catabolism exceeds the excretion^39^. The content of myo-inositol in human milk is highest in colostrum and decrease with time, however, the level seems to be consistent in mature milk^40^. The observed decrease in the urine myo-inositol level from 1 to 3 months align with the findings from Cesare Marincola et al.^41^. The decline could be due to higher catabolism of myo-inositol in the kidney and higher requirement to produce phosphatidylinositols, for the growing infant.

The observed increase in hippurate levels over time suggest potential maturation of the gut microbiota^42^. Bacteria produce hippurate from plant-derived phenols which originate from the diet of the mother^43,44^. Tartrate, related to the gut microbiome as bacteria degrade tartrate^45^, shows a decrease in levels over time, which could indicate a maturation of the gut microbiota.

Interestingly, some of the metabolites shown to alter during time in infant urine, also alters if the infant receives formula milk compared to breast milk. This could suggest that these metabolites are rather fluctuating^47^. A study by Giallourou et al. of urine metabolites uses the metabolite levels to determine the metabolic age of stunted children^11^. Our study contributes with useful knowledge to the field of urine metabolite profiling and can be used as reference values. We clearly observe a metabolic maturation during the first 3 months of life, but there are some inconsistencies with other studies for example do we observe an increase in citrate during time, while the other study observed a decrease^11^.

### Conserved metabolite levels within the individual

Not only is it interesting to look at the fixed effects and the relation to the metabolite levels. The random effects indicate which metabolites are conserved within the individual when not including the fixed effects. The four most conserved metabolites are glucose, taurine, methionine, and myo-inositol. Within these four metabolites we see the random effects between individuals, given by the individuals’ identifier (ID) are higher than the random effects within individuals, given by the residual, meaning that the metabolite level is repeated within the individual for example over time (Table 3). The standard deviations of the ID estimate and residuals are presented in Table 3. Values for all random effects are presented in Supplementary Table S2.

**Table 3:** Estimates of the random effects of the metabolites with a ratio > 1 and the four metabolites with the lowest ratio.

Metabolite	Standard deviation		
	ID	Residual	Ratio (ID^2^/residual^2^)
Glucose	0.174	0.154	1.270
Taurine	0.202	0.192	1.106
Methionine	0.018	0.018	1.057
Myo-inositol	0.209	0.206	1.027
Threonine	0.024	0.081	0.087
Valine	0.007	0.025	0.079
S-Adenosylhomcysteine	0.001	0.002	0.067
Acetate	0.152	0.595	0.066

ID: Identifier.

The four metabolites listed in Table 3 with a ratio > 1 are two sugars; glucose and myo-inositol and two amino acids; taurine and methionine. However, why these metabolites are conserved across time and not the others are difficult to explain. A similar trend was observed for the fecal metabolome, where a high intra-individual variability was observed^48^. Another reason for the conserved levels of the metabolites could be due to genetics. Some studies indicate that the basal metabolic rate is partly determined by genotype, which could mean that metabolites that take part in the energy processes like glucose and myo-inositol will be affected by the genetics^49^. On the contrary, acetate, s-adenosylhomocysteine, threonine, and valine are the metabolites which show the lowest ratio, meaning they have the lowest conservation in the individual over time (Table 3).

### The metabolic maturation age

As described above, a strong age signature was observed in the urine metabolome of the infants. We utilized this age signature to estimate a metabolic urine age as a surrogate of metabolic maturation. The metabolic age was predicted using a two component PLS model with sample age in days as the response variable and metabolite levels as the explanatory variables. The cumulated goodness of fit (R2Y) and the cumulated predictive accuracy (Q2Y) of the PLS model were 0.60 and 0.56, respectively. The root mean squared error of cross validation (RMSECV) was 16.5 days.

**Figure 3:**
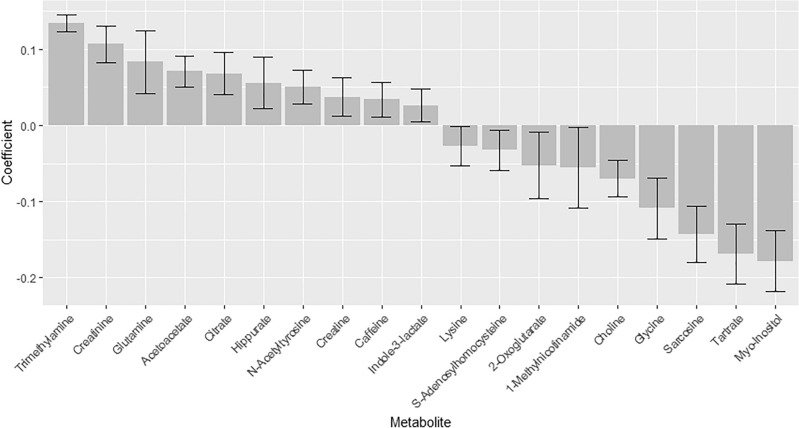
Bar plot of metabolite coefficients with confidence intervals from a PLS model of urine sample age and metabolite levels. Grey bars: metabolite coefficient, whiskers: confidence intervals.

A bar plot is presented in Fig. 3 showing the significant (p < 0.05) metabolites in the model, which are characteristic for the metabolic urine age. A table with coefficients and confidence intervals of all metabolites can be found in Supporting information (Table S3). It is observed that a high metabolic urine age is associated with at low amount of lysine, s-adenosylhomocysteine, sarcosine, 2-oxogluterate, 1-methylnicotinamide, choline, glycine, sarcosine, tartrate, and myo-inositol, in contrary to trimethylamine, creatinine, glutamine, acetoacetate, citrate, hippurate, N-acetyltyrosine, creatine, caffeine, and indole-3-lactate, which are associated with high levels for an older urine sample, which agrees with the PCA model in Fig. 1.

The study correlated metabolic maturity with 1-year outcomes of weight and height in children, adjusting for gestational age, infant sex, and birth weight. The only significant finding was a negative association between weight at 1 year and metabolic maturity at 3 months with an estimate of − 0.025 (results shown in Table S4 in Supporting Information). A more mature metabolite profile at 3 months, corresponding to a higher predicted metabolic age, was linked to a lower 1-year weight.

When connecting this to the metabolites driving the model, this means that a lower weight at 1 year is associated with low urine levels of lysine, S-adenosylhomocysteine, sarcosine, 2-oxogluterate, 1-methylnicotinamide, choline, glycine, sarcosine, tartrate, and myo-inositol, and high levels of trimethylamine, creatinine, glutamine, acetoacetate, citrate, hippurate, N-acetyltyrosine, creatine, caffeine, and indole-3-lactateat 3 months. How these urine metabolite levels could have an impact on the weight at 1 year is difficult to determine with an observational study like this. The R2Y of 0.60 is the cumulated goodness of fit and is the variance explained by the model, hence 60% is explained while 40% is unexplained. Likewise, the Q2Y and RMSECV of 0.56 and 16.5 days indicate that some of the variation in the model is not described by the time, but potentially being attributed to biological differences in maturity pace among infants. In comparison to Giallourou et al.’s study using urine metabolites to determine metabolic age in older infants, our study identified different altered metabolites, possibly due to variations in infant growth due to age differences in the studied infants^11^. Our study's significant metabolites span various classes, including amino acids, energy-related compounds, fatty acids, food-derived substances, sugars, and unclassified compounds. The lack of an apparent pattern among these metabolites makes it challenging to explain why higher urine metabolic maturation is associated with lower weight and height outcomes at 1 year. The infant weight at 1 year is influenced by multiple factors including feeding practice^50^, gestational age, sex, and birth weight^51^. Our study, predominantly with breastfed infants, corrected for gestational age, sex, and birth weight. The contribution of the metabolic maturation determined from urine metabolites related to the metabolic processes which could affect the infant weight at 1 year are discussed below.

The unexpected inverse relationship between higher metabolic maturation age and lower weight at 1 year could indicate that swift passage through the early maturation stage may hinder optimal growth. This could be because the infant struggle at sustaining some of the metabolites like trimethylamine, creatinine, glutamine, and acetoacetate, leading to their elevated levels in urine (Fig. 3). As described above, glutamine takes part in the protein and nucleotide synthesis, and a higher excretion could potentially lead to decreased production of important proteins and nucleotides important for the development of the child. Similarly, increased acetoacetate, which is an important energy source for the heart muscle, could impact overall energy balance. Conversely, lower amounts of allantoin, glycine, s-adenosylhomocysteine (SAH), sarcosine, tartrate, myo-inositol, and 1-methylnicotinamide in the 3-month urine of the children with lower 1-year weight may signify altered metabolism. Myo-inositol, crucial for anabolic processes and act as growth mediator^52^, might be excessively retained, potentially influencing later growth at 1 year. Interestingly, SAH, precursor of homocysteine and constituent in the methionine cycle^53^, was not significantly altered in linear mixed effects model analysis of the urine levels from 1 to 3 months of age. It is crucial to note that assumptions about high weight correlating with a more mature/developed child need careful consideration. A too high weight is associated with risk of overweight later in life^54^ and it is therefore important that the infant/child weight follows a normal growth rate.

## Strengths and limitations

This study is, to our knowledge, the largest investigation of the infant urine metabolome in the initial 3 months of life, using repeated sampling from the same infant. This approach minimizes bias and provides distinct insight into dynamic metabolic changes and enables examination of fluctuating and stable metabolites in the infant during the initial months. The PLS model showed the feasibility of predicting the metabolic maturation age based on the measured metabolites. This should, however, be done with caution due to the uncertainty of the model reflected in the cumulated R2Y, Q2Y and RMSECV of 0.60, 0.56, and 16.5 days, respectively. It is essential to acknowledge the potential impact of uncontrolled factors in sample collection. Despite given instructions to avoid sampling bias, the samples were collected in the participants’ own home, which could introduce uncertainties of for example variation in collection times. Furthermore, some biological variance is expected which could potentially be mitigated by expanding sample size. A limitation of the study is the approx. 10% attrition rate in sample collection, which raises concerns about potential bias associated with sample compliance. However, a comparative analysis of the participant characteristics across the three age-stages shows no significant differences. The study, thereby, underline the importance of controlled sampling conditions and the consideration of potential biases in interpreting the predictive capabilities of the model.

## Conclusion

The current study provides reference values of the metabolites in the healthy infant urine at 1, 2, and 3 months. The results from the PCA and linear mixed-effects models showed significant changes from 1 to 3 months in 15 of the 49 measured metabolites. After Bonferroni-correction we only observed significant β-estimates related to age-stage. One important result is the significant change of urine creatinine from 1 to 3 months as creatinine often is used as normalization for other urine metabolite levels. Our results indicate that care should be taken when using this normalization strategy for infants. The linear mixed-effects models also revealed that some metabolites are more conserved in the infant than others, these included glucose, taurine, methionine, and myo-inositol. Lastly, the PLS model based on urine sample age and metabolite level was used to predict a metabolic maturation urine age. The metabolic maturation age at 3 months was significantly associated with the weight of the child at 1 year. In conclusion, the present study adds knowledge to the field of metabolic changes and maturation that takes place in the infant during the first 3 months of life.

### Supplementary Information

Supplementary Information.

## Data Availability

The data from this study is available upon request from the corresponding authors due to ongoing sample collection and analysis.
