# Supplementary material for: Metabolic maturation in the infant urine during the first 3 months of life
Source: Sci Rep. 2024 Mar 8;14:5697. doi: 10.1038/s41598-024-56227-7 (PMC10924096; doi:10.1038/s41598-024-56227-7)
Supplement: Supplementary file 1 — Supplementary Information. [file 41598_2024_56227_MOESM1_ESM.pdf]

Title: Metabolic maturation in the infant urine during the first three months of life

Author List: Julie Astono, Katrine O. Poulsen, Rikke A. Larsen, Emma V. Jessen, Chatrine B. Sand, Morten A. Rasmussen, Ulrik K. Sundekilde

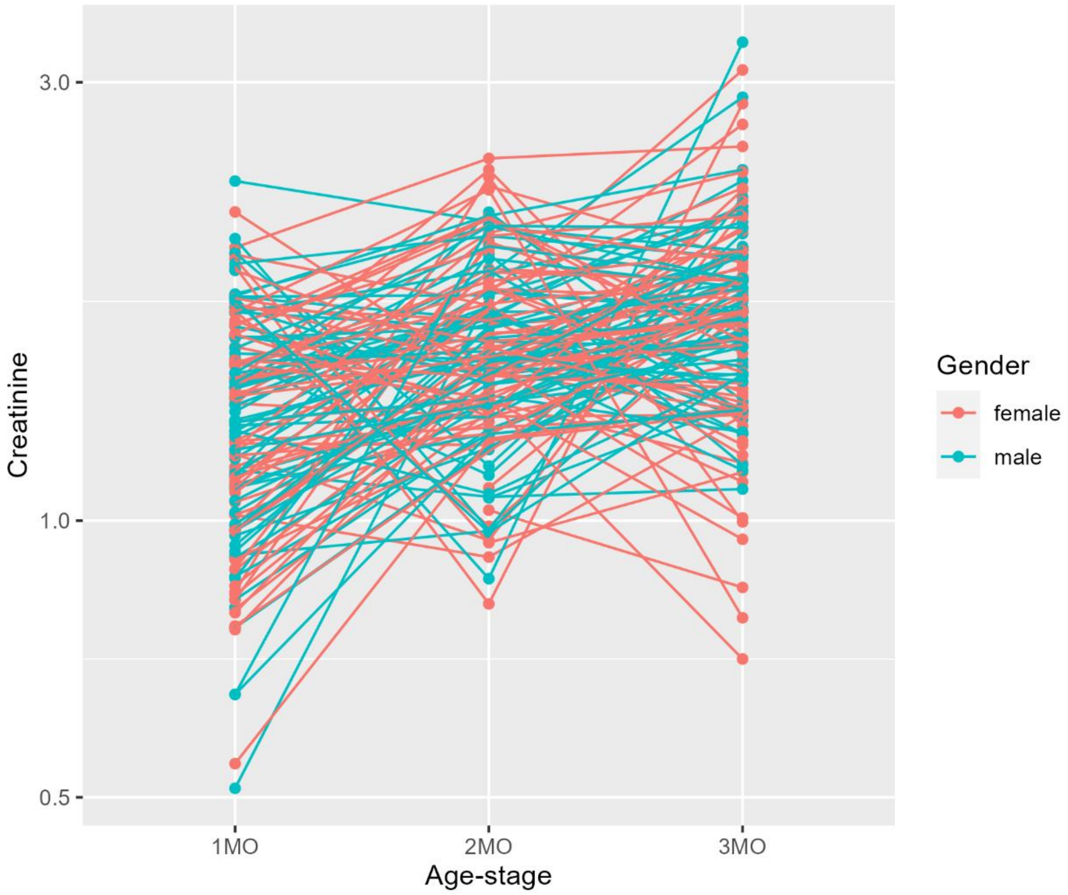

Figure S1. Creatinine levels for each sample according to time of sampling. Coloured according to gender.

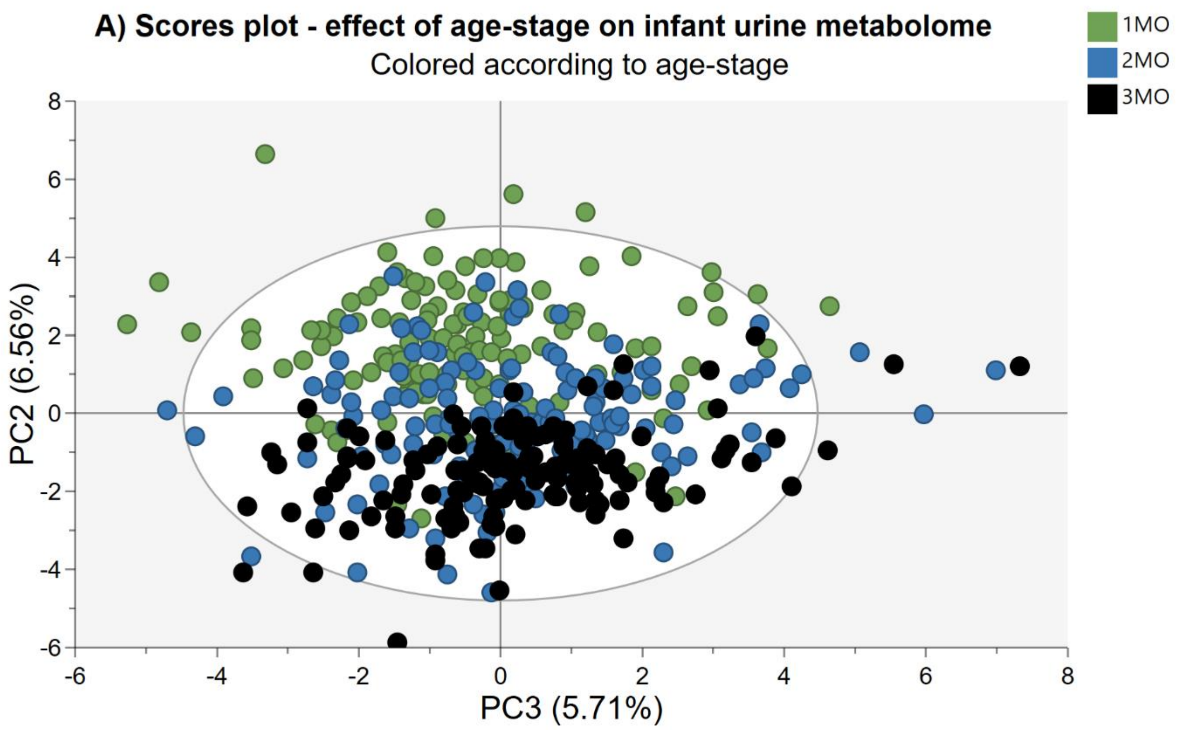

Figure S2. Scores plot of principal component 3 on the x-axis and principal component 2 on y-axis of samples colored according to age-stage.

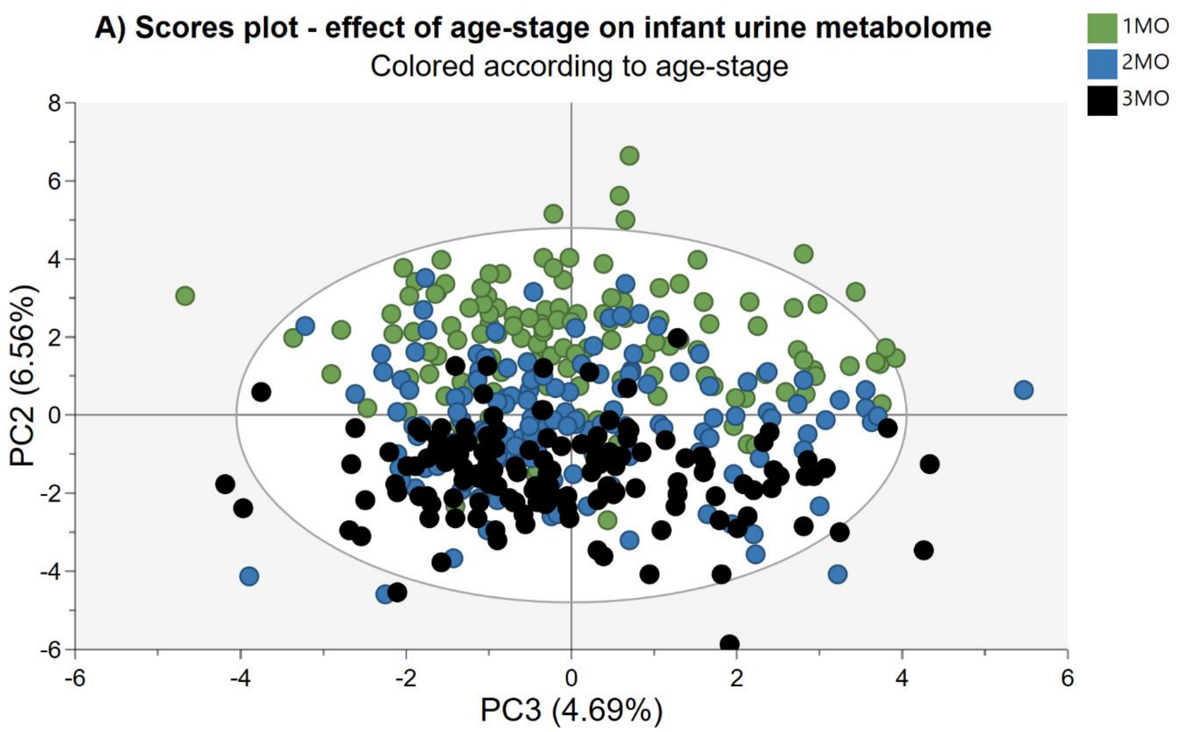

Figure S3. Scores plot of principal component 4 on the x-axis and principal component 2 on y-axis of samples colored according to age-stage.

Table S1. Results from all fixed effects terms of the linear mixed-effects models including β-estimate, standard deviation, p-value and q-value (p-value after Bonferroni correction)

**Metabolite**	Fixed effect	**Estimate**	Standard deviation	**p-value**	**q**-value
**1-Methylnicotinamide**	From one to two months	-0.0160	0.0053	0.0029	1.0000
**2-Oxoglutarate**	From one to two months	-0.0477	0.0136	0.0005	0.2024
**Acetate**	From one to two months	0.0772	0.0714	0.2807	1.0000
**Acetoacetate**	From one to two months	0.0016	0.0010	0.0903	1.0000
**Acetone**	From one to two months	-0.0034	0.0022	0.1329	1.0000
**Alanine**	From one to two months	0.0164	0.0084	0.0508	1.0000
**Allantoin**	From one to two months	0.0013	0.0023	0.5844	1.0000
**Betaine**	From one to two months	0.0341	0.0385	0.3759	1.0000
**Caffeine**	From one to two months	0.0043	0.0038	0.2605	1.0000
**Carnitine**	From one to two months	0.0023	0.0032	0.4653	1.0000
**Choline**	From one to two months	-0.0120	0.0025	0.0000	0.0010
**Citrate**	From one to two months	0.1679	0.0899	0.0628	1.0000
**Creatine**	From one to two months	0.0025	0.0150	0.8684	1.0000
**Creatinine**	From one to two months	0.2498	0.0429	0.0000	0.0000
**Cysteine**	From one to two months	-0.0264	0.0127	0.0376	1.0000
**Dimethylamine**	From one to two months	0.0140	0.0115	0.2239	1.0000
**Formate**	From one to two months	0.0169	0.0733	0.8173	1.0000
**Fumarate**	From one to two months	-0.0024	0.0008	0.0023	0.8967
**Galactose**	From one to two months	-0.0442	0.0345	0.2002	1.0000
**Glucose**	From one to two months	-0.0105	0.0188	0.5752	1.0000
**Glutamine**	From one to two months	0.0794	0.0115	0.0000	0.0000
**Glutathione**	From one to two months	-0.0031	0.0017	0.0692	1.0000
**Glycine**	From one to two months	-0.2587	0.0400	0.0000	0.0000
**Guanidoacetate**	From one to two months	-0.0022	0.0073	0.7678	1.0000
**Hippurate**	From one to two months	0.0187	0.0096	0.0530	1.0000
**Homocysteine**	From one to two months	0.0338	0.0273	0.2157	1.0000
**Indole-3-lactate**	From one to two months	0.0023	0.0021	0.2924	1.0000
**Lactate**	From one to two months	0.0137	0.0395	0.7298	1.0000
**Lactose**	From one to two months	-0.0121	0.0267	0.6518	1.0000
**Lysine**	From one to two months	0.0003	0.0044	0.9380	1.0000
**Mannose**	From one to two months	-0.0033	0.0044	0.4435	1.0000
**Methionine**	From one to two months	-0.0025	0.0021	0.2383	1.0000
**Myo-Inositol**	From one to two months	-0.3988	0.0252	0.0000	0.0000
**N,N-Dimethylglycine**	From one to two months	-0.0318	0.0073	0.0000	0.0083
**N-Acetyltyrosine**	From one to two months	0.0221	0.0058	0.0002	0.0691
**O-Phosphoethanolamine**	From one to two months	-0.0056	0.0047	0.2394	1.0000
**Pantothenate**	From one to two months	0.0024	0.0017	0.1708	1.0000
**Phenylacetate**	From one to two months	0.0064	0.0036	0.0715	1.0000
**Propylene glycol**	From one to two months	0.0658	0.1746	0.7065	1.0000
**Pyruvate**	From one to two months	-0.0036	0.0034	0.2841	1.0000

**S-Adenosylhomocysteine**	From one to two months	-0.0002	0.0003	0.5014	1.0000
**Sarcosine**	From one to two months	-0.0038	0.0004	0.0000	0.0000
**Serine**	From one to two months	0.0482	0.0194	0.0138	1.0000
**Succinate**	From one to two months	-0.0046	0.0053	0.3926	1.0000
**Tartrate**	From one to two months	-0.0206	0.0019	0.0000	0.0000
**Taurine**	From one to two months	0.0369	0.0234	0.1162	1.0000
**Threonine**	From one to two months	-0.0065	0.0098	0.5082	1.0000
**Trimethylamine**	From one to two months	0.0014	0.0002	0.0000	0.0000
**Valine**	From one to two months	0.0015	0.0030	0.6147	1.0000
**1-Methylnicotinamide**	From one to three months	-0.0238	0.0054	0.0000	0.0057
**2-Oxoglutarate**	From one to three months	-0.0672	0.0138	0.0000	0.0008
**Acetate**	From one to three months	-0.0222	0.0724	0.7597	1.0000
**Acetoacetate**	From one to three months	0.0053	0.0010	0.0000	0.0000
**Acetone**	From one to three months	-0.0044	0.0023	0.0545	1.0000
**Alanine**	From one to three months	0.0101	0.0085	0.2378	1.0000
**Allantoin**	From one to three months	-0.0060	0.0024	0.0119	1.0000
**Betaine**	From one to three months	-0.0415	0.0391	0.2902	1.0000
**Caffeine**	From one to three months	0.0110	0.0038	0.0044	1.0000
**Carnitine**	From one to three months	0.0061	0.0033	0.0626	1.0000
**Choline**	From one to three months	-0.0179	0.0025	0.0000	0.0000
**Citrate**	From one to three months	0.5650	0.0914	0.0000	0.0000
**Creatine**	From one to three months	0.0567	0.0152	0.0002	0.0922
**Creatinine**	From one to three months	0.3941	0.0435	0.0000	0.0000
**Cysteine**	From one to three months	0.0053	0.0129	0.6834	1.0000
**Dimethylamine**	From one to three months	-0.0062	0.0116	0.5967	1.0000
**Formate**	From one to three months	0.0322	0.0744	0.6653	1.0000
**Fumarate**	From one to three months	-0.0016	0.0008	0.0401	1.0000
**Galactose**	From one to three months	-0.0403	0.0350	0.2503	1.0000
**Glucose**	From one to three months	-0.0134	0.0191	0.4846	1.0000

**Glutamine**	From one to three months	0.0969	0.0117	0.0000	0.0000
**Glutathione**	From one to three months	0.0024	0.0017	0.1624	1.0000
**Glycine**	From one to three months	-0.4235	0.0406	0.0000	0.0000
**Guanidoacetate**	From one to three months	-0.0023	0.0074	0.7592	1.0000
**Hippurate**	From one to three months	0.0425	0.0098	0.0000	0.0076
**Homocysteine**	From one to three months	0.0715	0.0277	0.0102	1.0000
**Indole-3-lactate**	From one to three months	0.0060	0.0022	0.0060	1.0000
**Lactate**	From one to three months	-0.0271	0.0402	0.4997	1.0000
**Lactose**	From one to three months	-0.0481	0.0271	0.0773	1.0000
**Lysine**	From one to three months	-0.0121	0.0045	0.0075	1.0000
**Mannose**	From one to three months	-0.0153	0.0044	0.0006	0.2512
**Methionine**	From one to three months	-0.0062	0.0022	0.0045	1.0000
**Myo-Inositol**	From one to three months	-0.6131	0.0256	0.0000	0.0000
**N,N-Dimethylglycine**	From one to three months	-0.0314	0.0075	0.0000	0.0140
**N-Acetyltyrosine**	From one to three months	0.0282	0.0059	0.0000	0.0011
**O-Phosphoethanolamine**	From one to three months	-0.0077	0.0048	0.1124	1.0000
**Pantothenate**	From one to three months	-0.0014	0.0017	0.4237	1.0000
**Phenylacetate**	From one to three months	0.0032	0.0036	0.3791	1.0000
**Propylene glycol**	From one to three months	0.1135	0.1773	0.5225	1.0000
**Pyruvate**	From one to three months	-0.0062	0.0034	0.0716	1.0000
**S-Adenosylhomocysteine**	From one to three months	-0.0007	0.0003	0.0133	1.0000
**Sarcosine**	From one to three months	-0.0064	0.0004	0.0000	0.0000
**Serine**	From one to three months	0.0286	0.0197	0.1489	1.0000
**Succinate**	From one to three months	-0.0060	0.0054	0.2713	1.0000
**Tartrate**	From one to three months	-0.0342	0.0019	0.0000	0.0000

**Taurine**	From one to three months	0.0429	0.0239	0.0734	1.0000
**Threonine**	From one to three months	-0.0243	0.0100	0.0152	1.0000
**Trimethylamine**	From one to three months	0.0025	0.0002	0.0000	0.0000
**Valine**	From one to three months	-0.0018	0.0030	0.5527	1.0000
**1-Methylnicotinamide**	From two to three months	-0.0078	0.0053	0.1382	1.0000
**2-Oxoglutarate**	From two to three months	-0.0195	0.0134	0.1474	1.0000
**Acetate**	From two to three months	-0.0993	0.0709	0.1622	1.0000
**Acetoacetate**	From two to three months	0.0037	0.0009	0.0001	0.0476
**Acetone**	From two to three months	-0.0010	0.0022	0.6471	1.0000
**Alanine**	From two to three months	-0.0063	0.0083	0.4429	1.0000
**Allantoin**	From two to three months	-0.0073	0.0023	0.0018	0.7074
**Betaine**	From two to three months	-0.0756	0.0381	0.0481	1.0000
**Caffeine**	From two to three months	0.0068	0.0037	0.0712	1.0000
**Carnitine**	From two to three months	0.0038	0.0032	0.2380	1.0000
**Choline**	From two to three months	-0.0059	0.0025	0.0179	1.0000
**Citrate**	From two to three months	0.3971	0.0889	0.0000	0.0046
**Creatine**	From two to three months	0.0542	0.0148	0.0003	0.1161
**Creatinine**	From two to three months	0.1443	0.0425	0.0008	0.3092
**Cysteine**	From two to three months	0.0317	0.0125	0.0117	1.0000
**Dimethylamine**	From two to three months	-0.0201	0.0114	0.0777	1.0000
**Formate**	From two to three months	0.0153	0.0725	0.8330	1.0000
**Fumarate**	From two to three months	0.0008	0.0008	0.3210	1.0000
**Galactose**	From two to three months	0.0039	0.0341	0.9084	1.0000
**Glucose**	From two to three months	-0.0028	0.0185	0.8779	1.0000
**Glutamine**	From two to three months	0.0176	0.0113	0.1224	1.0000

**Glutathione**	From two to three months	0.0055	0.0017	0.0012	0.4678
**Glycine**	From two to three months	-0.1647	0.0395	0.0000	0.0160
**Guanidoacetate**	From two to three months	-0.0001	0.0072	0.9869	1.0000
**Hippurate**	From two to three months	0.0238	0.0095	0.0134	1.0000
**Homocysteine**	From two to three months	0.0377	0.0270	0.1641	1.0000
**Indole-3-lactate**	From two to three months	0.0038	0.0021	0.0760	1.0000
**Lactate**	From two to three months	-0.0408	0.0392	0.2983	1.0000
**Lactose**	From two to three months	-0.0360	0.0264	0.1740	1.0000
**Lysine**	From two to three months	-0.0124	0.0044	0.0047	1.0000
**Mannose**	From two to three months	-0.0120	0.0043	0.0059	1.0000
**Methionine**	From two to three months	-0.0037	0.0021	0.0798	1.0000
**Myo-Inositol**	From two to three months	-0.2143	0.0248	0.0000	0.0000
**N,N-Dimethylglycine**	From two to three months	0.0004	0.0072	0.9592	1.0000
**N-Acetyltyrosine**	From two to three months	0.0062	0.0057	0.2842	1.0000
**O-Phosphoethanolamine**	From two to three months	-0.0021	0.0047	0.6583	1.0000
**Pantothenate**	From two to three months	-0.0038	0.0017	0.0273	1.0000
**Phenylacetate**	From two to three months	-0.0033	0.0035	0.3556	1.0000
**Propylene glycol**	From two to three months	0.0477	0.1729	0.7828	1.0000
**Pyruvate**	From two to three months	-0.0026	0.0034	0.4438	1.0000
**S-Adenosylhomocysteine**	From two to three months	-0.0005	0.0003	0.0630	1.0000
**Sarcosine**	From two to three months	-0.0026	0.0004	0.0000	0.0000
**Serine**	From two to three months	-0.0196	0.0193	0.3096	1.0000
**Succinate**	From two to three months	-0.0014	0.0053	0.7908	1.0000
**Tartrate**	From two to three months	-0.0136	0.0019	0.0000	0.0000
**Taurine**	From two to three months	0.0059	0.0231	0.7972	1.0000

**Threonine**	From two to three months	-0.0178	0.0098	0.0686	1.0000
**Trimethylamine**	From two to three months	0.0012	0.0002	0.0000	0.0001
**Valine**	From two to three months	-0.0033	0.0030	0.2659	1.0000
**1-Methylnicotinamide**	Birth weight (g)	0.0000	0.0000	0.0347	1.0000
**2-Oxoglutarate**	Birth weight (g)	0.0000	0.0000	0.2357	1.0000
**Acetate**	Birth weight (g)	0.0000	0.0001	0.5169	1.0000
**Acetoacetate**	Birth weight (g)	0.0000	0.0000	0.2220	1.0000
**Acetone**	Birth weight (g)	0.0000	0.0000	0.9067	1.0000
**Alanine**	Birth weight (g)	0.0000	0.0000	0.7447	1.0000
**Allantoin**	Birth weight (g)	0.0000	0.0000	0.8624	1.0000
**Betaine**	Birth weight (g)	0.0000	0.0001	0.7902	1.0000
**Caffeine**	Birth weight (g)	0.0000	0.0000	0.1809	1.0000
**Carnitine**	Birth weight (g)	0.0000	0.0000	0.7437	1.0000
**Choline**	Birth weight (g)	0.0000	0.0000	0.0239	1.0000
**Citrate**	Birth weight (g)	0.0000	0.0001	0.8482	1.0000
**Creatine**	Birth weight (g)	0.0000	0.0000	0.8839	1.0000
**Creatinine**	Birth weight (g)	0.0001	0.0000	0.0248	1.0000
**Cysteine**	Birth weight (g)	0.0000	0.0000	0.7871	1.0000
**Dimethylamine**	Birth weight (g)	0.0000	0.0000	0.8365	1.0000
**Formate**	Birth weight (g)	0.0000	0.0001	0.9280	1.0000
**Fumarate**	Birth weight (g)	0.0000	0.0000	0.5070	1.0000
**Galactose**	Birth weight (g)	0.0000	0.0000	0.2916	1.0000
**Glucose**	Birth weight (g)	0.0000	0.0000	0.8136	1.0000
**Glutamine**	Birth weight (g)	0.0000	0.0000	0.6897	1.0000
**Glutathione**	Birth weight (g)	0.0000	0.0000	0.5918	1.0000
**Glycine**	Birth weight (g)	-0.0001	0.0001	0.3993	1.0000
**Guanidoacetate**	Birth weight (g)	0.0000	0.0000	0.7259	1.0000
**Hippurate**	Birth weight (g)	0.0000	0.0000	0.4814	1.0000
**Homocysteine**	Birth weight (g)	0.0000	0.0000	0.8652	1.0000
**Indole-3-lactate**	Birth weight (g)	0.0000	0.0000	0.9421	1.0000
**Lactate**	Birth weight (g)	0.0000	0.0000	0.3212	1.0000
**Lactose**	Birth weight (g)	0.0000	0.0000	0.8478	1.0000
**Lysine**	Birth weight (g)	0.0000	0.0000	0.4966	1.0000
**Mannose**	Birth weight (g)	0.0000	0.0000	0.7200	1.0000
**Methionine**	Birth weight (g)	0.0000	0.0000	0.0969	1.0000
**Myo-Inositol**	Birth weight (g)	-0.0001	0.0000	0.0429	1.0000
**N,N-Dimethylglycine**	Birth weight (g)	0.0000	0.0000	0.0318	1.0000
**N-Acetyltyrosine**	Birth weight (g)	0.0000	0.0000	0.0274	1.0000
**O-Phosphoethanolamine**	Birth weight (g)	0.0000	0.0000	0.3285	1.0000
**Pantothenate**	Birth weight (g)	0.0000	0.0000	0.3822	1.0000
**Phenylacetate**	Birth weight (g)	0.0000	0.0000	0.6376	1.0000
**Propylene glycol**	Birth weight (g)	0.0001	0.0002	0.7649	1.0000

**Pyruvate**	Birth weight (g)	0.0000	0.0000	0.7797	1.0000
**S-Adenosylhomocysteine**	Birth weight (g)	0.0000	0.0000	0.3052	1.0000
**Sarcosine**	Birth weight (g)	0.0000	0.0000	0.0004	0.1375
**Serine**	Birth weight (g)	0.0000	0.0000	0.1264	1.0000
**Succinate**	Birth weight (g)	0.0000	0.0000	0.4758	1.0000
**Tartrate**	Birth weight (g)	0.0000	0.0000	0.0160	1.0000
**Taurine**	Birth weight (g)	0.0000	0.0000	0.3195	1.0000
**Threonine**	Birth weight (g)	0.0000	0.0000	0.4963	1.0000
**Trimethylamine**	Birth weight (g)	0.0000	0.0000	0.1499	1.0000
**Valine**	Birth weight (g)	0.0000	0.0000	0.1925	1.0000
**1-Methylnicotinamide**	C-section(No to yes)	-0.0001	0.0090	0.9891	1.0000
**2-Oxoglutarate**	C-section(No to yes)	0.0381	0.0342	0.2669	1.0000
**Acetate**	C-section(No to yes)	-0.0841	0.1100	0.4462	1.0000
**Acetoacetate**	C-section(No to yes)	0.0000	0.0015	0.9787	1.0000
**Acetone**	C-section(No to yes)	-0.0031	0.0042	0.4673	1.0000
**Alanine**	C-section(No to yes)	-0.0628	0.0225	0.0060	1.0000
**Allantoin**	C-section(No to yes)	-0.0009	0.0046	0.8518	1.0000
**Betaine**	C-section(No to yes)	0.0469	0.0795	0.5557	1.0000
**Caffeine**	C-section(No to yes)	-0.0087	0.0081	0.2816	1.0000
**Carnitine**	C-section(No to yes)	0.0027	0.0069	0.6996	1.0000
**Choline**	C-section(No to yes)	0.0115	0.0061	0.0601	1.0000
**Citrate**	C-section(No to yes)	0.3740	0.1911	0.0523	1.0000
**Creatine**	C-section(No to yes)	-0.0210	0.0334	0.5303	1.0000
**Creatinine**	C-section(No to yes)	-0.1173	0.0723	0.1069	1.0000
**Cysteine**	C-section(No to yes)	0.0005	0.0315	0.9877	1.0000
**Dimethylamine**	C-section(No to yes)	-0.0011	0.0192	0.9529	1.0000
**Formate**	C-section(No to yes)	0.0576	0.1492	0.7001	1.0000
**Fumarate**	C-section(No to yes)	0.0036	0.0020	0.0738	1.0000
**Galactose**	C-section(No to yes)	-0.0203	0.0658	0.7580	1.0000
**Glucose**	C-section(No to yes)	-0.0069	0.0561	0.9024	1.0000
**Glutamine**	C-section(No to yes)	-0.0238	0.0276	0.3900	1.0000
**Glutathione**	C-section(No to yes)	0.0006	0.0029	0.8479	1.0000
**Glycine**	C-section(No to yes)	0.0059	0.0908	0.9480	1.0000
**Guanidoacetate**	C-section(No to yes)	-0.0064	0.0150	0.6701	1.0000
**Hippurate**	C-section(No to yes)	0.0030	0.0166	0.8595	1.0000
**Homocysteine**	C-section(No to yes)	-0.0172	0.0464	0.7118	1.0000
**Indole-3-lactate**	C-section(No to yes)	-0.0051	0.0050	0.3054	1.0000
**Lactate**	C-section(No to yes)	-0.0072	0.0727	0.9211	1.0000
**Lactose**	C-section(No to yes)	0.0163	0.0517	0.7526	1.0000
**Lysine**	C-section(No to yes)	-0.0100	0.0099	0.3148	1.0000
**Mannose**	C-section(No to yes)	-0.0019	0.0097	0.8481	1.0000
**Methionine**	C-section(No to yes)	-0.0100	0.0060	0.0948	1.0000
**Myo-Inositol**	C-section(No to yes)	0.1180	0.0693	0.0908	1.0000
**N,N-Dimethylglycine**	C-section(No to yes)	0.0373	0.0196	0.0595	1.0000

**N-Acetyltyrosine**	C-section(No to yes)	-0.0085	0.0118	0.4714	1.0000
**O-Phosphoethanolamine**	C-section(No to yes)	-0.0109	0.0091	0.2328	1.0000
**Pantothenate**	C-section(No to yes)	-0.0032	0.0045	0.4742	1.0000
**Phenylacetate**	C-section(No to yes)	0.0021	0.0087	0.8077	1.0000
**Propylene glycol**	C-section(No to yes)	-0.2214	0.3276	0.5004	1.0000
**Pyruvate**	C-section(No to yes)	-0.0027	0.0054	0.6171	1.0000
**S-Adenosylhomocysteine**	C-section(No to yes)	-0.0009	0.0004	0.0481	1.0000
**Sarcosine**	C-section(No to yes)	0.0008	0.0010	0.3942	1.0000
**Serine**	C-section(No to yes)	0.0079	0.0339	0.8171	1.0000
**Succinate**	C-section(No to yes)	-0.0211	0.0099	0.0356	1.0000
**Tartrate**	C-section(No to yes)	-0.0006	0.0041	0.8864	1.0000
**Taurine**	C-section(No to yes)	-0.0155	0.0664	0.8155	1.0000
**Threonine**	C-section(No to yes)	-0.0263	0.0155	0.0922	1.0000
**Trimethylamine**	C-section(No to yes)	-0.0004	0.0004	0.2633	1.0000
**Valine**	C-section(No to yes)	-0.0085	0.0047	0.0707	1.0000
**1-Methylnicotinamide**	Gestational age (weeks)	0.0000	0.0005	0.9278	1.0000
**2-Oxoglutarate**	Gestational age (weeks)	0.0001	0.0021	0.9553	1.0000
**Acetate**	Gestational age (weeks)	0.0019	0.0066	0.7707	1.0000
**Acetoacetate**	Gestational age (weeks)	0.0001	0.0001	0.3180	1.0000
**Acetone**	Gestational age (weeks)	-0.0003	0.0003	0.3104	1.0000
**Alanine**	Gestational age (weeks)	-0.0005	0.0014	0.7286	1.0000
**Allantoin**	Gestational age (weeks)	0.0001	0.0003	0.7231	1.0000
**Betaine**	Gestational age (weeks)	0.0110	0.0048	0.0233	1.0000
**Caffeine**	Gestational age (weeks)	-0.0005	0.0005	0.2807	1.0000
**Carnitine**	Gestational age (weeks)	0.0009	0.0004	0.0349	1.0000
**Choline**	Gestational age (weeks)	0.0004	0.0004	0.2552	1.0000
**Citrate**	Gestational age (weeks)	0.0061	0.0115	0.5982	1.0000
**Creatine**	Gestational age (weeks)	0.0031	0.0020	0.1236	1.0000
**Creatinine**	Gestational age (weeks)	-0.0011	0.0043	0.7933	1.0000
**Cysteine**	Gestational age (weeks)	0.0014	0.0019	0.4752	1.0000
**Dimethylamine**	Gestational age (weeks)	0.0008	0.0012	0.4913	1.0000
**Formate**	Gestational age (weeks)	-0.0231	0.0090	0.0113	1.0000
**Fumarate**	Gestational age (weeks)	0.0001	0.0001	0.5546	1.0000
**Galactose**	Gestational age (weeks)	-0.0003	0.0040	0.9307	1.0000
**Glucose**	Gestational age (weeks)	0.0030	0.0034	0.3817	1.0000
**Glutamine**	Gestational age (weeks)	-0.0004	0.0017	0.8009	1.0000
**Glutathione**	Gestational age (weeks)	-0.0001	0.0002	0.6372	1.0000
**Glycine**	Gestational age (weeks)	-0.0010	0.0055	0.8582	1.0000
**Guanidoacetate**	Gestational age (weeks)	0.0003	0.0009	0.7101	1.0000
**Hippurate**	Gestational age (weeks)	0.0000	0.0010	0.9659	1.0000
**Homocysteine**	Gestational age (weeks)	-0.0012	0.0028	0.6642	1.0000
**Indole-3-lactate**	Gestational age (weeks)	0.0001	0.0003	0.8531	1.0000
**Lactate**	Gestational age (weeks)	0.0047	0.0044	0.2854	1.0000
**Lactose**	Gestational age (weeks)	0.0022	0.0031	0.4869	1.0000

**Lysine**	Gestational age (weeks)	-0.0002	0.0006	0.7671	1.0000
**Mannose**	Gestational age (weeks)	0.0002	0.0006	0.6716	1.0000
**Methionine**	Gestational age (weeks)	-0.0005	0.0004	0.1971	1.0000
**Myo-Inositol**	Gestational age (weeks)	0.0052	0.0042	0.2158	1.0000
**N,N-Dimethylglycine**	Gestational age (weeks)	0.0007	0.0012	0.5707	1.0000
**N-Acetyltyrosine**	Gestational age (weeks)	-0.0001	0.0007	0.8464	1.0000
**O-Phosphoethanolamine**	Gestational age (weeks)	0.0004	0.0005	0.5062	1.0000
**Pantothenate**	Gestational age (weeks)	0.0003	0.0003	0.2389	1.0000
**Phenylacetate**	Gestational age (weeks)	0.0005	0.0005	0.3401	1.0000
**Propylene glycol**	Gestational age (weeks)	-0.0132	0.0197	0.5038	1.0000
**Pyruvate**	Gestational age (weeks)	0.0004	0.0003	0.1774	1.0000
**S-Adenosylhomocysteine**	Gestational age (weeks)	0.0000	0.0000	0.5404	1.0000
**Sarcosine**	Gestational age (weeks)	0.0000	0.0001	0.5617	1.0000
**Serine**	Gestational age (weeks)	-0.0026	0.0020	0.2108	1.0000
**Succinate**	Gestational age (weeks)	-0.0003	0.0006	0.5979	1.0000
**Tartrate**	Gestational age (weeks)	0.0003	0.0002	0.1613	1.0000
**Taurine**	Gestational age (weeks)	0.0035	0.0040	0.3874	1.0000
**Threonine**	Gestational age (weeks)	-0.0024	0.0009	0.0125	1.0000
**Trimethylamine**	Gestational age (weeks)	0.0000	0.0000	0.5689	1.0000
**Valine**	Gestational age (weeks)	-0.0003	0.0003	0.3470	1.0000
**1-Methylnicotinamide**	BMI	0.0002	0.0005	0.6754	1.0000
**2-Oxoglutarate**	BMI	-0.0020	0.0019	0.2967	1.0000
**Acetate**	BMI	0.0077	0.0061	0.2099	1.0000
**Acetoacetate**	BMI	0.0000	0.0001	0.7895	1.0000
**Acetone**	BMI	-0.0004	0.0002	0.0984	1.0000
**Alanine**	BMI	0.0009	0.0012	0.4864	1.0000
**Allantoin**	BMI	0.0000	0.0003	0.9806	1.0000
**Betaine**	BMI	-0.0052	0.0044	0.2342	1.0000
**Caffeine**	BMI	-0.0007	0.0004	0.1465	1.0000
**Carnitine**	BMI	0.0005	0.0004	0.2053	1.0000
**Choline**	BMI	-0.0003	0.0003	0.4152	1.0000
**Citrate**	BMI	0.0056	0.0105	0.5994	1.0000
**Creatine**	BMI	0.0043	0.0018	0.0224	1.0000
**Creatinine**	BMI	-0.0051	0.0040	0.2079	1.0000
**Cysteine**	BMI	0.0038	0.0017	0.0310	1.0000
**Dimethylamine**	BMI	0.0005	0.0011	0.6477	1.0000
**Formate**	BMI	-0.0061	0.0082	0.4611	1.0000
**Fumarate**	BMI	0.0000	0.0001	0.8991	1.0000
**Galactose**	BMI	-0.0006	0.0036	0.8727	1.0000
**Glucose**	BMI	0.0002	0.0031	0.9382	1.0000
**Glutamine**	BMI	0.0015	0.0015	0.3096	1.0000
**Glutathione**	BMI	0.0000	0.0002	0.7682	1.0000
**Glycine**	BMI	0.0097	0.0050	0.0547	1.0000
**Guanidoacetate**	BMI	0.0008	0.0008	0.3538	1.0000

**Hippurate**	BMI	0.0003	0.0009	0.7196	1.0000
**Homocysteine**	BMI	0.0041	0.0026	0.1157	1.0000
**Indole-3-lactate**	BMI	-0.0001	0.0003	0.7906	1.0000
**Lactate**	BMI	0.0092	0.0040	0.0242	1.0000
**Lactose**	BMI	-0.0016	0.0029	0.5843	1.0000
**Lysine**	BMI	0.0001	0.0005	0.8432	1.0000
**Mannose**	BMI	0.0008	0.0005	0.1603	1.0000
**Methionine**	BMI	0.0000	0.0003	0.9218	1.0000
**Myo-Inositol**	BMI	0.0001	0.0038	0.9816	1.0000
**N,N-Dimethylglycine**	BMI	0.0003	0.0011	0.7933	1.0000
**N-Acetyltyrosine**	BMI	-0.0002	0.0007	0.7159	1.0000
**O-Phosphoethanolamine**	BMI	0.0004	0.0005	0.4842	1.0000
**Pantothenate**	BMI	-0.0001	0.0002	0.7571	1.0000
**Phenylacetate**	BMI	0.0000	0.0005	0.9451	1.0000
**Propylene glycol**	BMI	-0.0216	0.0181	0.2343	1.0000
**Pyruvate**	BMI	0.0004	0.0003	0.2013	1.0000
**S-Adenosylhomocysteine**	BMI	0.0000	0.0000	0.9818	1.0000
**Sarcosine**	BMI	0.0000	0.0001	0.9721	1.0000
**Serine**	BMI	0.0018	0.0019	0.3448	1.0000
**Succinate**	BMI	0.0004	0.0005	0.4967	1.0000
**Tartrate**	BMI	0.0000	0.0002	0.8653	1.0000
**Taurine**	BMI	-0.0103	0.0036	0.0052	1.0000
**Threonine**	BMI	-0.0007	0.0009	0.4422	1.0000
**Trimethylamine**	BMI	0.0000	0.0000	0.5528	1.0000
**Valine**	BMI	0.0004	0.0003	0.1051	1.0000
**1-Methylnicotinamide**	Gender (female to male)	-0.0077	0.0053	0.1530	1.0000
**2-Oxoglutarate**	Gender (female to male)	-0.0359	0.0200	0.0756	1.0000
**Acetate**	Gender (female to male)	0.0154	0.0653	0.8140	1.0000
**Acetoacetate**	Gender (female to male)	0.0000	0.0009	0.9861	1.0000
**Acetone**	Gender (female to male)	-0.0026	0.0025	0.2926	1.0000
**Alanine**	Gender (female to male)	0.0256	0.0132	0.0536	1.0000
**Allantoin**	Gender (female to male)	0.0012	0.0027	0.6684	1.0000
**Betaine**	Gender (female to male)	-0.0603	0.0468	0.1998	1.0000
**Caffeine**	Gender (female to male)	-0.0095	0.0048	0.0469	1.0000
**Carnitine**	Gender (female to male)	-0.0063	0.0041	0.1205	1.0000
**Choline**	Gender (female to male)	-0.0062	0.0036	0.0847	1.0000
**Citrate**	Gender (female to male)	0.1796	0.1124	0.1123	1.0000
**Creatine**	Gender (female to male)	-0.0455	0.0196	0.0220	1.0000
**Creatinine**	Gender (female to male)	-0.0032	0.0428	0.9398	1.0000
**Cysteine**	Gender (female to male)	-0.0156	0.0185	0.3987	1.0000
**Dimethylamine**	Gender (female to male)	-0.0152	0.0114	0.1823	1.0000
**Formate**	Gender (female to male)	0.0135	0.0879	0.8779	1.0000
**Fumarate**	Gender (female to male)	-0.0004	0.0012	0.7272	1.0000
**Galactose**	Gender (female to male)	0.0818	0.0389	0.0369	1.0000

**Glucose**	Gender (female to male)	-0.0379	0.0328	0.2498	1.0000
**Glutamine**	Gender (female to male)	0.0051	0.0162	0.7520	1.0000
**Glutathione**	Gender (female to male)	-0.0030	0.0017	0.0901	1.0000
**Glycine**	Gender (female to male)	0.0237	0.0534	0.6577	1.0000
**Guanidoacetate**	Gender (female to male)	0.0030	0.0088	0.7360	1.0000
**Hippurate**	Gender (female to male)	-0.0090	0.0099	0.3603	1.0000
**Homocysteine**	Gender (female to male)	-0.0019	0.0275	0.9449	1.0000
**Indole-3-lactate**	Gender (female to male)	-0.0007	0.0029	0.8123	1.0000
**Lactate**	Gender (female to male)	-0.0117	0.0429	0.7851	1.0000
**Lactose**	Gender (female to male)	-0.0258	0.0305	0.3986	1.0000
**Lysine**	Gender (female to male)	0.0029	0.0058	0.6225	1.0000
**Mannose**	Gender (female to male)	-0.0073	0.0057	0.2015	1.0000
**Methionine**	Gender (female to male)	0.0007	0.0035	0.8353	1.0000
**Myo-Inositol**	Gender (female to male)	-0.0314	0.0406	0.4396	1.0000
**N,N-Dimethylglycine**	Gender (female to male)	0.0124	0.0115	0.2839	1.0000
**N-Acetyltyrosine**	Gender (female to male)	0.0053	0.0069	0.4477	1.0000
**O-Phosphoethanolamine**	Gender (female to male)	-0.0032	0.0054	0.5543	1.0000
**Pantothenate**	Gender (female to male)	-0.0051	0.0026	0.0551	1.0000
**Phenylacetate**	Gender (female to male)	-0.0005	0.0051	0.9213	1.0000
**Propylene glycol**	Gender (female to male)	0.0508	0.1934	0.7932	1.0000
**Pyruvate**	Gender (female to male)	-0.0039	0.0032	0.2282	1.0000
**S-Adenosylhomocysteine**	Gender (female to male)	0.0000	0.0003	0.9335	1.0000
**Sarcosine**	Gender (female to male)	0.0004	0.0006	0.4800	1.0000
**Serine**	Gender (female to male)	-0.0284	0.0201	0.1597	1.0000
**Succinate**	Gender (female to male)	0.0005	0.0059	0.9380	1.0000
**Tartrate**	Gender (female to male)	0.0038	0.0024	0.1194	1.0000
**Taurine**	Gender (female to male)	-0.0545	0.0388	0.1626	1.0000
**Threonine**	Gender (female to male)	0.0113	0.0092	0.2215	1.0000
**Trimethylamine**	Gender (female to male)	0.0000	0.0002	0.8541	1.0000
**Valine**	Gender (female to male)	0.0059	0.0028	0.0343	1.0000

Table S2. Estimates of the standard deviation of random effects of the ID, residual and the ratio between them.

Metabolite	ID	Residual	Ratio ($ID^2/Residual^2$)
**1-Methylnicotinamide**	0.017	0.044	0.156
**2-Oxoglutarate**	0.099	0.112	0.793
**Acetate**	0.152	0.595	0.066
**Acetoacetate**	0.003	0.008	0.110
**Acetone**	0.010	0.018	0.279
**Alanine**	0.067	0.069	0.960
**Allantoin**	0.011	0.019	0.338
**Betaine**	0.203	0.318	0.410
**Caffeine**	0.021	0.031	0.472

**Carnitine**	0.018	0.026	0.473
**Choline**	0.017	0.021	0.719
**Citrate**	0.502	0.742	0.457
**Creatine**	0.091	0.123	0.545
**Creatinine**	0.138	0.356	0.150
**Cysteine**	0.091	0.104	0.766
**Dimethylamine**	0.036	0.095	0.143
**Formate**	0.378	0.605	0.389
**Fumarate**	0.006	0.006	0.870
**Galactose**	0.156	0.285	0.298
**Glucose**	0.174	0.154	1.280
**Glutamine**	0.078	0.095	0.688
**Glutathione**	0.006	0.014	0.186
**Glycine**	0.250	0.329	0.577
**Guanidoacetate**	0.038	0.060	0.399
**Hippurate**	0.034	0.080	0.178
**Homocysteine**	0.091	0.226	0.161
**Indole-3-lactate**	0.014	0.018	0.617
**Lactate**	0.163	0.328	0.248
**Lactose**	0.124	0.221	0.314
**Lysine**	0.027	0.036	0.543
**Mannose**	0.026	0.036	0.537
**Methionine**	0.018	0.018	1.057
**Myo-Inositol**	0.209	0.206	1.027
**N,N-Dimethylglycine**	0.058	0.060	0.941
**N-Acetyltyrosine**	0.030	0.048	0.383
**O-Phosphoethanolamine**	0.022	0.039	0.305
**Pantothenate**	0.013	0.014	0.893
**Phenylacetate**	0.025	0.029	0.717
**Propylene glycol**	0.757	1.445	0.274
**Pyruvate**	0.009	0.028	0.104
**S-Adenosylhomocysteine**	0.001	0.002	0.067
**Sarcosine**	0.003	0.003	0.707
**Serine**	0.070	0.161	0.187
**Succinate**	0.023	0.044	0.268
**Tartrate**	0.011	0.016	0.477
**Taurine**	0.202	0.192	1.106
**Threonine**	0.024	0.082	0.087
**Trimethylamine**	0.001	0.002	0.149
**Valine**	0.007	0.025	0.079

Table S3. Metabolite coefficients and the confidence intervals in the PLS model of urine sample age and metabolite level.

Metabolite	Coefficient	Confidence intervals

1-Methylnicotinamide	-0.056	0.053
2-Oxoglutarate	-0.053	0.043
Acetate	-0.002	0.045
Acetoacetate	0.071	0.021
Acetone	-0.026	0.032
Alanine	0.009	0.055
Allantoin	-0.034	0.037
Betaine	-0.018	0.055
Caffeine	0.034	0.022
Carnitine	0.016	0.040
Choline	-0.070	0.024
Citrate	0.068	0.028
Creatine	0.037	0.025
Creatinine	0.107	0.024
Cysteine	0.004	0.025
Dimethylamine	-0.009	0.032
Formate	0.005	0.060
Fumarate	-0.022	0.042
Galactose	-0.015	0.028
Glucose	-0.010	0.017
Glutamine	0.083	0.041
Glutathione	0.022	0.035
Glycine	-0.109	0.041
Guanidoacetate	-0.008	0.040
Hippurate	0.056	0.034
Homocysteine	0.032	0.052
Indole-3-lactate	0.026	0.022
Lactate	-0.007	0.029
Lactose	-0.020	0.039
Lysine	-0.027	0.026
Mannose	-0.038	0.044
Methionine	-0.023	0.027
N,N-Dimethylglycine	-0.039	0.047
N-Acetyltyrosine	0.050	0.022
O-Phosphoethanolamine	-0.021	0.043
Pantothenate	-0.012	0.046
Phenylacetate	0.010	0.024
Propylene glycol	0.010	0.033
Pyruvate	-0.022	0.044
S-Adenosylhomocysteine	-0.032	0.027
Sarcosine	-0.143	0.037
Serine	0.019	0.055
Succinate	-0.017	0.045
Tartrate	-0.169	0.040
Taurine	0.012	0.029
Threonine	-0.031	0.033

Trimethylamine	0.135	0.011
Valine	-0.006	0.039
Myo-Inositol	-0.178	0.040

Table S4. Results of the linear models with predicted metabolic urine age and prospective outcomes height and weight of the child at one year.

Outcome at one year	Age-stage	Estimate	p-value
**Height**	1 Month	-0.029	0.168
**Weight**	1 Month	-0.001	0.164
**Height**	2 Months	0.006	0.838
**Weight**	2 Months	0.003	0.668
**Height**	3 Months	-0.030	0.441
**Weight**	3 Months	-0.025	0.032
